# Catabolic profiling of selective enzymes in the saccharification of non-food lignocellulose parts of biomass into functional edible sugars and bioenergy: An *in silico* bioprospecting

**DOI:** 10.5455/javar.2022.i565

**Published:** 2022-01-14

**Authors:** Parag Kumar Paul, Salauddin Al Azad, Mohammad Habibur Rahman, Mithila Farjana, Muhammad Ramiz Uddin, Dipta Dey, Shafi Mahmud, Tanzila Ismail Ema, Partha Biswas, Maliha Anjum, Ozifatun Jannat Akhi, Shahlaa Zernaz Ahmed

**Affiliations:** 1 Department of Electrical and Electronic Engineering, United International University, Dhaka, Bangladesh; 2 Fermentation Engineering Major, School of Biotechnology, Jiangnan University, Wuxi, China; 3 Vaccinology Lab, Department of Microbiology and Hygiene, Faculty of Veterinary Science, Bangladesh Agricultural University, Mymensingh 2202, Bangladesh; 4 Department of Chemistry and Biochemistry, University of Oklahoma, Norman, OK, USA; 5 Department of Biochemistry and Molecular Biology, Bangabandhu Sheikh Mujibur Rahman Science and Technology University, Gopalganj, Bangladesh; 6 Department of Genetic Engineering and Biotechnology, University of Rajshahi, Rajshahi, Bangladesh; 7 Department of Biochemistry and Microbiology, North South University, Dhaka, Bangladesh; 8 Department of Genetic Engineering and Biotechnology, Jashore University of Science and Technology, Jashore, Bangladesh; 9 Department of Computer Science and Engineering, Mymensingh Engineering College, Mymensingh, Bangladesh

**Keywords:** Catabolic profiling, enzymatic hydrolysis, lignocellulose biomass, saccharification, functional edible sugars, biofuel, molecular dynamic simulation

## Abstract

**Objectives:**

The research aims to analyze the catabolic strength of different hydrolytic enzymes in assessing the biological conversion potential of lignocellulose parts of agricultural biomass wastes into functional edible sugars and biofuels.

**Materials and Methods:**

The enzymes’ hydrolytic properties—versatile peroxidase, manganese peroxidase, and lignin peroxidase were used to identify their complexing strength with the lignin substrate, whereas endoglucanase cel12A, acidocaldarius cellulase, and Melanocarpus albomyces endoglucanase were tested on the cellulose gel substrate. Because the biodegradation properties are heavily influenced by the “enzyme-substrate complexing energy level,” proper molecular optimization and energy minimization of the enzymes and substrates were carried out, as well as the identification of the enzyme’s active sites prior to complexing.comprehensive molecular dynamic simulation was run to study their—alpha carbon, root-mean-square deviation (*Å*), molecular surface area (*Å2*), root-mean-square fluctuation (*Å*), radius of gyration (nm), hydrogen bonds with hydrophobic interactions, and solvent accessible surface area (*Å2*) values for 50 ns. The simulated data mining was conducted using advanced programming algorithms to establish the final enzyme-substrate complexing strength in binding and catalysis.

**Results:**

Among the lignin-degrading enzymes, versatile peroxidase shows promising catalytic activity with the best docking pose and significant values in all the dynamic simulation parameters. Similarly, Melanocarpus albomyces endoglucanase shows the best activity in all aspects of molecular docking and dynamics among the cellulose-degrading enzymes.

**Conclusion:**

The lignin content of biomass wastes can be degraded into cellulose and hemicellulose using lignin-degrading enzymes. The cellulose can be further degraded into glucose and xylose sugars following the cellulose-degrading enzyme activity. These sugars can be further degraded into biofuel through anaerobic fermentation. Systematic bioconversion of the lignocellulosic components can ensure sustainable biomass management, creating an alternative food and energy source for human beings to face the challenges of global hunger where the enzymes can pave the way.

## Introduction

The world’s population has quadrupled over the last century [1]. Lignocellulosic biomass has typically been utilized to produce bioethanol, a novel and renewable energy resource for bioethanol production as a sustainable substitute for fossil fuels and toxic chemicals [6]. However, by utilizing the catabolic activities of several enzymes, this biomass can be converted into glucose and xylose [7] as the edible energy sources of humans. First, lignocellulosic biomass can be enzymatically hydrolyzed to expose the unstable cellulose and hemicellulose portions. Second, cellulose can be further catabolized to produce glucose and xylose [8]. The total process is termed an enzyme-induced saccharification reaction [9].

Manganese peroxidase is a well-known lignin oxidizing and depolymerizing peroxidase, produced by soil fungi and basidiomycetes, causing white rot [10], which oxidizes the lignocellulose biomass containing manganese (II) ions (Mn2+) into reactive Mn3+. This Mn3+ is stabilized by chelators such as oxalic acid, which is found in fungus and bacteria. Stabilized Mn3+ attacks phenolic lignin to disintegrate lignocellulosic biomass spontaneously [11]. Other enzymes of the lignin-degrading family are versatile peroxidase and lignin peroxidase, which possess the unique characteristics of lignin degradation without the use of redox mediators [12]. As reported by established research, the hemicellulose is degraded by cellulases such as acidocaldarius cellulase, Melanocarpus albomyces endoglucanase, and standard endoglucanase cel12A. These cellulases catalyze the disintegration of hemicellulose by hydrolyzing the β-1,4-glycosidic bonds [13].

Considering these factors, the present study aims to analyze the activity of selective industrial enzymes in biosynthesizing edible glucose and xylose to mitigate the global food and energy demand in the near future. At the same time, it is predicted that the catabolic properties of enzymes can enable human beings to use lignocellulose biomass as a novel alternative source of edible sugars as well as biofuel, depending on their molecular docking and dynamic simulation profiles. 

## Materials and Methods 

### Extraction of the targeted substrates and enzymes 

Based on a comprehensive literature review, two substrates, lignin (PubChem CID-73555271) and cellulose microcrystal (PubChem CID-14055602), were selected in the current research. PubChem (https://pubchem.ncbi.nlm.nih.gov/), an National Center for Biotechnology Information authorized open chemistry repository, has been utilized to screen substrates based on in-depth analysis of their physicochemical properties for interacting with enzymes.

On the other hand, lignin peroxidase Protein Data Bank (PDB ID-1LLP), manganese peroxidase (PDB ID-1YYD), and versatile peroxidase (PDB ID-2BOQ) were considered as the main lignin-degrading enzymes, whereas endoglucanase cel12A (PDB ID-1H0B), Acidocaldarius cellulase (PDB ID-3GZK), and Melanocarpus albomyces endoglucanase (PDB ID-1OA9) were selected for cellulose gel (microcrystal) bio-degradation analysis. The set of enzymes was prepared using PDB (http://www.rcsb.org/pdb/). 

### Enzymes’ active site selection for supra-molecular docking pose identification 

The active sites of the selective enzymes were identified to ensure supramolecular docking with their corresponding substrates (lignin and cellulose gel) rather than conventional docking [14]. To ensure the best-fit regions of the enzymes to be docked with the substrates, core algorithm (COACH-D) (https://yanglab.nankai.edu.cn/COACH-D/) was used, following the suggested algorithm [15], so that the binding energy (kcal/mol), residue numbers, and the binding pose can be determined simultaneously. 

### Optimization of the substrates

**Figure 1.**
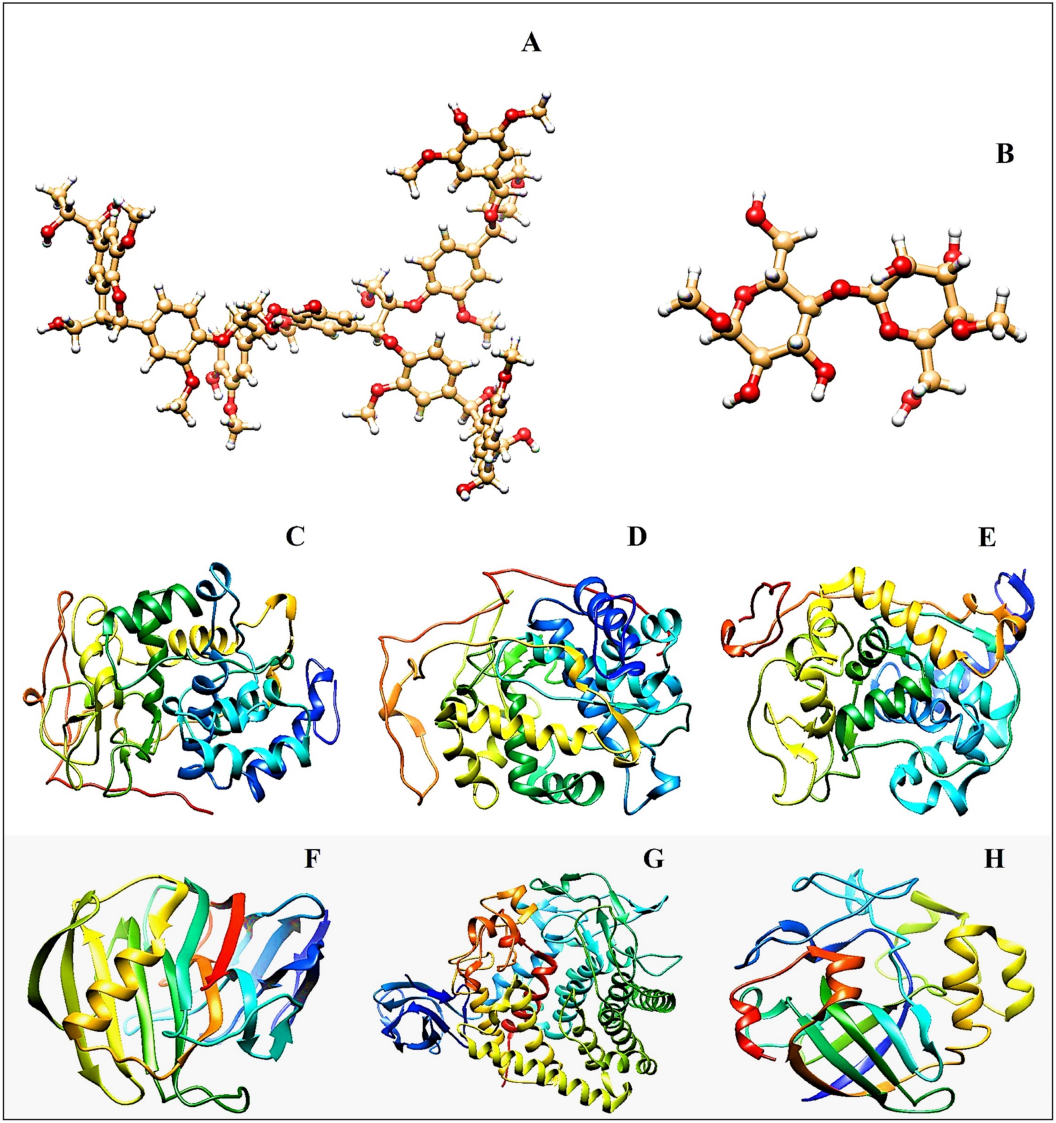
Illustration of the targeted carbohydrate structures, namely lignin and cellulose with the hydrolytic enzymes targeting them, means- Lignin Peroxidase (1LLP), Manganese Peroxidase (1YYD), and Versatile Peroxidase (2BOQ) for lignin; while Endoglucanase cel12A (1H0B), Cellulase CelA (3GZK), and Endoglucanase (1OA9) for cellulose. Optimized X-ray crystallographic structures of Lignin (A) and Cellulose (B). The lignin-degrading optimized enzymes are ILLP (C), 1YYD (D), and 2BOQ (E). In addition, the cellulose-degrading optimized enzymes are 1H0B (F), 3GZK (G), and 1OA9 (H).

The 3D structures of the selected substrates (lignin and cellulose gel) were procured in structure data file format from PubChem [16]. Energy minimization was done by reducing the accumulative charge on substrates to zero based on the Gasteiger and Marsili method (1978) and by using the University of California San Francisco Chimera (UCSF) Chimera Software Package (version-1.14) (https://www.cgl.ucsf.edu/chimera/) [17]. Following optimization, the substrates of interest were transformed into a “mol2 file” for further assessment and molecular docking. All the optimized structures of the substrates have been shown in (Fig. 1A and 1B). 

### Optimization of the enzymes

The enzymes were optimized sequentially, considering the specific chain selection of the enzymes, the removal of peripheral metal ions, co-factors, water molecules, heteroatoms, additional chains, and non-bonded substrate subunits that were interconnecting with the amino acid residues were made. Besides, the addition of hydrogen atoms and the minimization of energy were conducted due to point-specific docking. The optimization process was also conducted with the UCSF Chimera Software (version-1.14) (https://www.cgl.ucsf.edu/chimera/) [18]. All optimized enzymes were conserved (.pdb format) for the next steps [19]. The lignin and cellulose gel hydrolytic enzymes are presented in Figure 1, as sequenced-1LLP (C), 1YYD (D), 2B0Q (E), 1H0B (F), 3GZK (G), and 1OA9 (H). 

**Figure 2.**
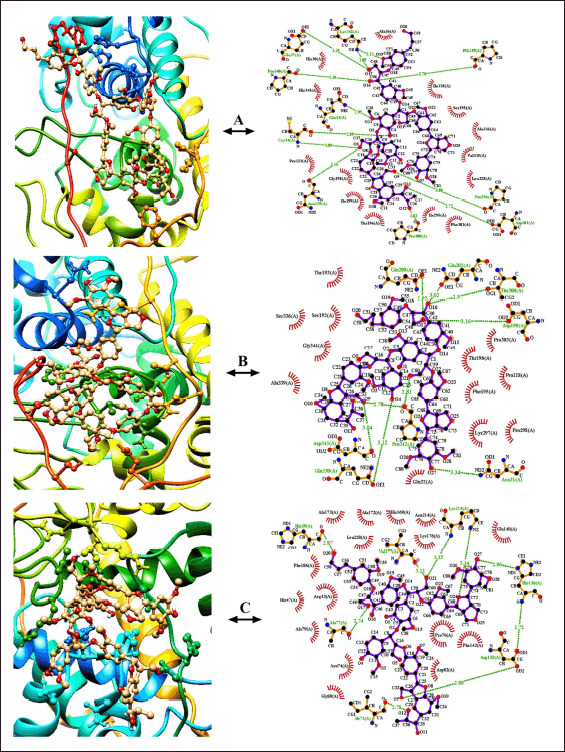
Diagrammatic representation of the enzyme-substrate dockings with their hydrogen bond interactions and hydrophobic interactions among each other. The docking profiles and hydrophobic interactive configurations of lignin with the hydrolytic enzymes- 1LLP (A); 1YYD (B), and 2BOQ (C), respectively, have been illustrated side by side.

### Operation of molecular docking 

Depending on the positions of the point-specific residues of the enzymes, point-specific super docking was conducted between the optimized substrates and their corresponding enzymes to examine the probable enzyme-substrate binding interactions using PyRx 0.8 docking software [20]. Here, the selected enzyme-substrate complex was changed into the “pdbqt format”. Following the supramolecular docking operation, the binding affinity of the substrates was calculated, and root mean square deviation (RMSD) was saved in the “CSV file” for each of the enzyme-substrate complexes.

### Post molecular docking analysis

The visualization software DS Visualizer (https://accelrys-discovery-studio-visualizer.software.informer.com/3.0/) was used for the initial visualization of enzyme-substrate interactions. Afterward, PyMOL version 2.4.1 (https://pymol.org/) was used to analyze and visualize the interactions developed. The enzyme-substrate complexes were saved as “PDB files” from PyMOL for further analysis. Finally, the saved PDB files were analyzed in Ligplot+ (version 2.2) (https://www.ebi.ac.uk/thornton-srv/software/LigPlus/) for secondary and quantitative networking of interactions (based on the *Java Runtime Environment interface*) in aspects of marking the potential hydrogen bonding interactions and scopes for non-covalent bond formation [21]. 

### Molecular dynamics simulation (MDS_50ns_)

First, the MDS of the enzymes were run on CABS-flex 2.0 (http://biocomp.chem.uw.edu.pl/CABSflex2) for 10 ns to observe its natural changes in structural orientation and the interactions with adjacent water molecules and ions [22]. Afterward, MDS for individual enzyme-substrate complexes were run on the Substrate and Receptor Molecular Dynamics tools (http://chemyang.ccnu.edu.cn/ccb/server/LARMD/index.php) up to 3.1 ns for an initial assessment of RMSD, root mean square fluctuation (RMSF), solvent-accessible surface area (SASA), principle component analysis, and Debye–Waller factor for thermostability (B-Factor) [23]. 

**Figure 3.**
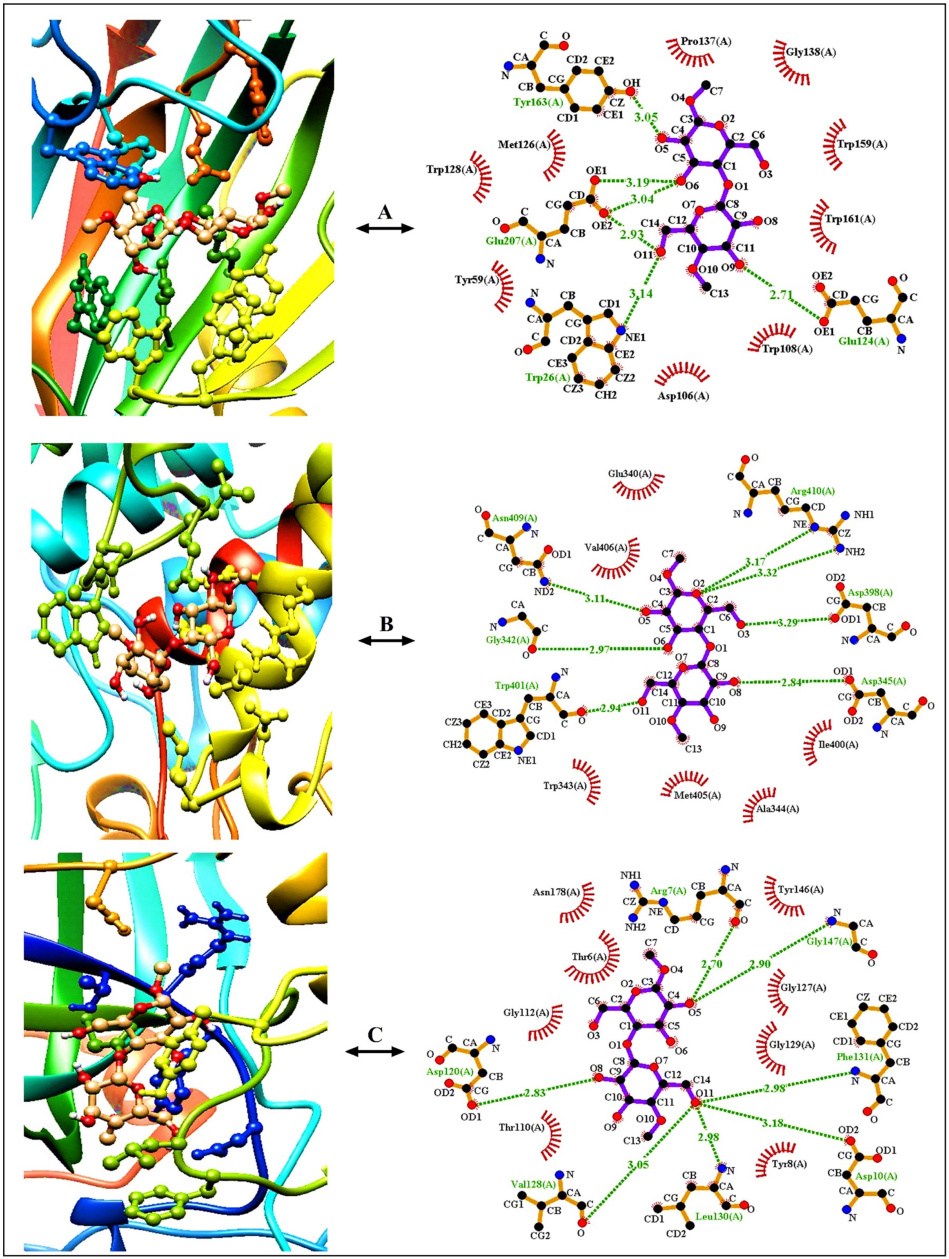
Diagrammatic representation of the enzyme-substrate dockings with their hydrogen bond interactions and hydrophobic interactions. The docking profiles and hydrophobic interactive configurations of cellulose with the hydrolytic enzymes- 1H0B (A), 3GZK (B), and 1OA9 (C), respectively, have been illustrated.

**Figure 4.**
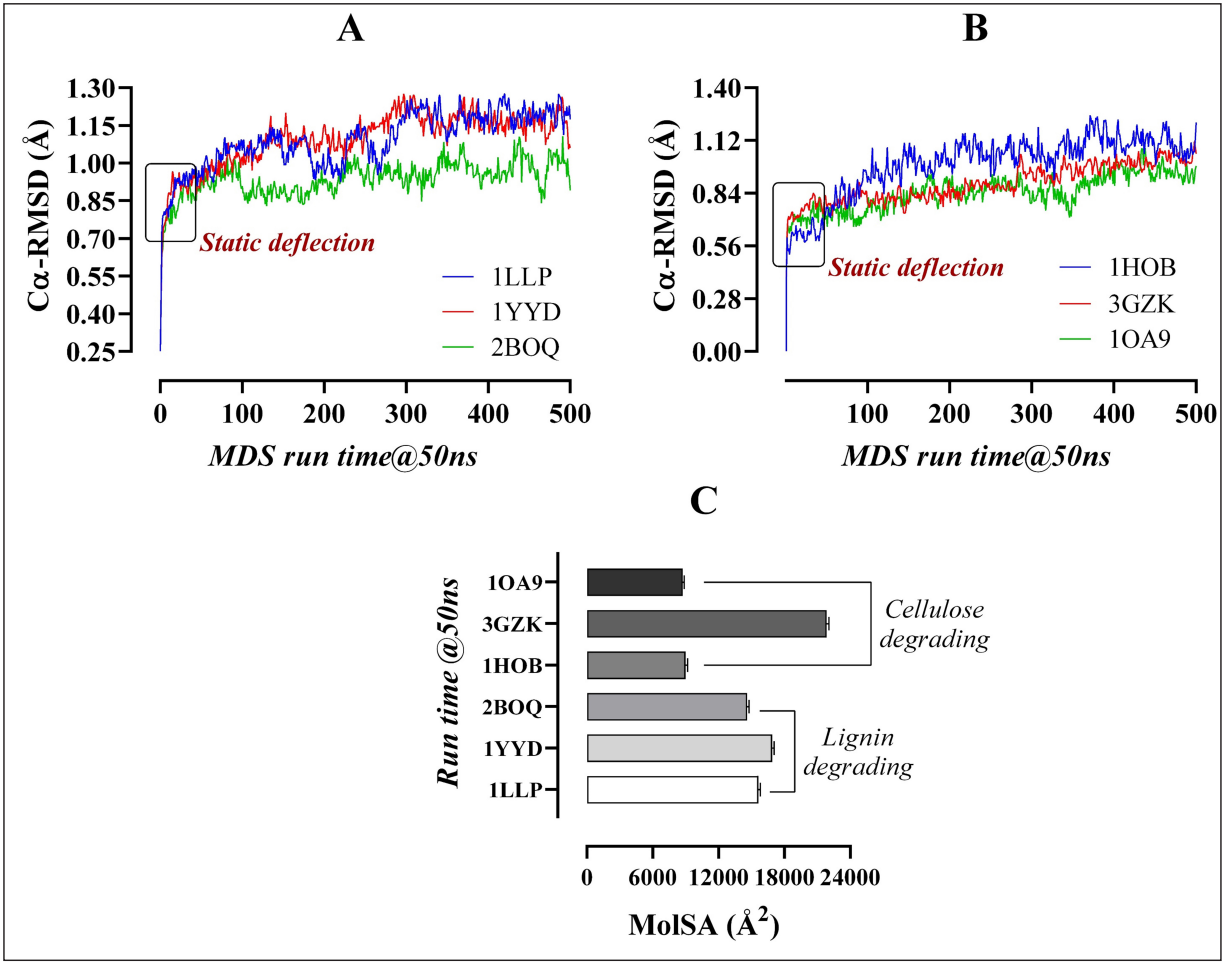
Analysis of the Cα-RMSD (*Å*) values obtained from the MDS (50ns) of both the lignin-degrading (A) and cellulose-degrading (B) enzymes. In addition, the MolSA values for all the enzymes, complexed with their corresponding substrates, are mentioned accordingly (C).

Finally, the comprehensive MDS of the biological complexes was executed in yet another scientific artificial reality application (YASARA) (version 11.9.18) to confirm the docking pattern and stability of various interactions [24]. The assisted model building with energy refinement-14 force field was used in this system, and the complexes were cleaned at the start of the experiment, and the network of hydrogen bonds was enhanced, and the water molecules were added. Besides, 0.9% NaCl was supplemented at a 310 K temperature, maintaining a pH level of 7.4 [25]. To evaluate the electrostatic-interactions, Particle Mesh Ewald’s method was applied [26]. The artificial cell was set at 20*Å*, which was bigger than all the biological complexes combined. This ensured free movement. The normal simulation time step of 1.25 fs was applied, and each simulation trajectory was conserved after a 100 ps interval. The simulation was run for 50 ns to analyze Cα-RMSD, Å, RMSF, Å, SASA, (Å^2^), the radius of gyration (Rg, nm), hydrogen bond, and molecular surface area (MolSA, Å^2^) [19,21].

### Statistical assessment of the resulting data

The data generated from the MDS for RMSD, RMSF, SASA, MolSA, and Rg were statistically analyzed using the “R programming language” (version R-4.0.2, for Linux) [27,28] and GraphPad Prism (version 8.0.1) [29–31]. The enzyme-substrate complexes were refined and graphically visualized using the aforementioned software packages.

## Results

### Enzymes’ active site selection for point-specific super docking 

In the enzymes’ active site selection process, a diversified number of amino acid residues were found in the target points (ranged between 9 and 27), depending on the enzyme types. Besides, distinguishable binding energy (kcal/mol) levels were predicted, considering the number of residues on the binding domains of the enzymes (Table 1). 

### Catabolism of the lignin-degrading enzymes based on structural interactions 

The lignin binding energy of the optimized enzymes 1LLP, 1YYD, and 2BOQ were −7.0; −6.5, and −8.0 (kcal/mol), respectively (Table 2). “1LLP-lignin” complex (Fig. 2A) contains total nine hydrogen bonds interactions with Pro339 (2.70 Å), Pro296 (3.00 Å), Asp201 (2.72 Å), Asn119 (3.15 Å), Cys34 (2.89 and 3.09 Å), Gln33 (2.95 Å), Pro340 (3.19 Å), Glu37 (3.29 Å) and Lys342 (3.19 and 3.09 Å); along with 14 hydrophobic interacting residues means- Ala36, IIe338, Ser195, Ala336, Val335, Leu328, Phe303, IIe295, Thn196, IIe199, Gly198, Pro121, His341, His30 (Table 2). Similarly, “1YYD-lignin” complex (Fig. 2B) is stabilized with eight hydrogen bonds such as Gln200 (2.92 Å), Gln302 (3.02 Å), Thr300 (2.97 Å), Asp198 (3.16 Å), Asn31 (3.34 Å), Gln330 (3.12 Å), Pro342 (2.81 and 2.78 Å) and Asp343 (3.04 Å). The 12 hydrophobic interactive residues were Pro303, Thr196, Pro118, Phe195, Lys297, Pro298, Glu32, Ala339, Gly344, Ser336, Ser192, and Thr193 (Table 2). In addition, the interaction between the “2BOQ-lignin” complex (Fig. 2C) was established on seven H-bond interactions, namely- Lys215 (3.15 and 3.14 Å), Vall77 (3.11 Å), His136 (2.96 and 2.72 Å), Asp135 (2.72 and 2.88 Å), IIe71 (2.78 Å), Ala77 (2.74 Å), and His39 (2.87 Å), as well as 16 hydrophobic interactions (Alal73, Alal72, His169, Leu228, Asn214, Lys176, Glu140, Pro76, Phe142, Asp82, Gly80, Asn74, Ala79, Arg43, His47, and Phe186) (Table 3). 

**Table 1.** The active site residues of amino acid of the enzymes used in this research with their positions and binding energy to the intended substrates (kcal/mol).

Enzyme names	Active positions of the amino acid residues	Binding energy (kcal/mol)	Super-docking points of the enzymes
**1LLP**	63, 66, 67, 69, 70, 73, 171, 172, 173, 180, 184, 198, 199, 201, 202, 204, 205, 206, 207, 208, 209, 210, 219, 261, 263, 291, 295	−10.3	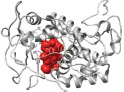
**1YYD**	56, 59, 60, 62, 63, 66, 163, 164, 165, 172, 176, 190, 191, 193, 194, 196, 197, 198, 199, 200, 201, 202, 211, 260, 262, 290, 294	−10.6	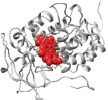
**2B0Q**	47, 58, 60, 92, 112, 113, 114, 218, 231, 232	−5.0	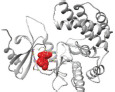
**1H0B**	44, 79, 142, 144, 161, 163, 198, 202, 204, 249	−7.1	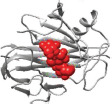
**3GZK**	143, 144, 146, 150, 221, 298, 300, 401, 515, 519, 520	−5.6	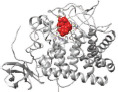
**1OA9**	10, 12, 13, 15, 18, 21, 45, 110, 131	−6.1	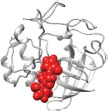

The red bubbles annotate the amino acid residue clusters in the enzymes’ active site.

**Figure 5.**
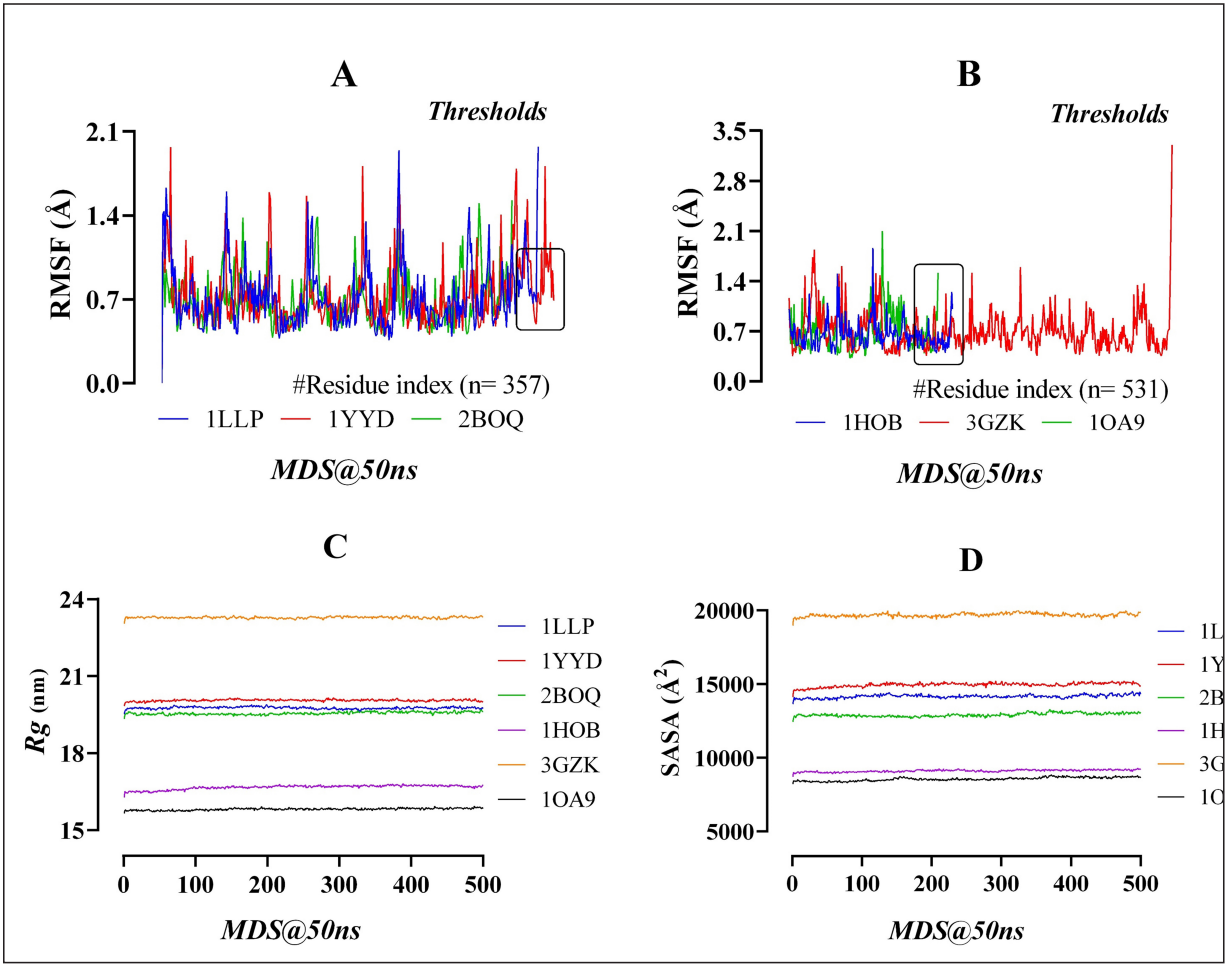
The RMSF (*Å*) profiling of the lignin (*A*), and cellulose (*B*) degrading enzymes resulted from the MDS (50 ns), along with their Rg (nm) and SASA (*Å^2^*), remarked as (C) and (D), respectively.

The MDS of 50 ns run time revealed that the RMSD values of the “Cα atom stability” of all complexes ranged between 0.8 and 1.25 Å. The “2BOQ-lignin” complex showed a better fluctuation rate between 30 and 50 ns time-period than the resting complexes (Fig. 4A). The MolSA (Å^2^) of the enzyme-substrate complexes was scored between 15,202.69 and 17,497.95 Å^2^ (Fig. 4C) for the lignin hydrolytic enzymes. Analysis of each enzyme’s flexibility resulted in RMSF values of less than 2 Å, indicating the enzymes’ more stable nature and a lower number of alpha-helix and beta-sheet entities (Fig. 5A) at the structural interaction points. The Rg (nm) values represent the tightness of the protein structure, where a lower degree of fluctuation stands for the significant uniformity and rigidity of the system. Among the three complexes, the 2BOQ-lignin complex showed the more compact structure following the value of 19.50, while the other complexes showed values as −19.80 (1YYD-lignin) and 19.60 (1LLP-lignin), respectively, which are less significant than the 2BOQ-lignin complexes (Fig. 5C). Besides, SASA confirmed the expansion of the enzymatic volume during the 50ns dynamics simulation process, where the “2BOQ-lignin” complex showed an effective output greater than the other complexes and represented the significant SASA value at 12,600 Å^2^. On the other hand, enzyme complexes such as 1LLP-lignin and 1YYD-lignin demonstrated higher SASA values at 140,002 and 145,002, respectively. Notably, the lowest SASA value of the system displayed more significant results that were found from the 2BOQ-lignin interaction (Fig. 5D). Among the lignin-degrading enzymes, 1LLP displayed polar and apolar energy per area at 4,592.05 and 9,123.59, respectively, whereas 1YYD produces polar energy at 5,226.47 and apolar energy at 9,473.63. Last but not least, 2BOQ presents values of 4,401.29 and 8,374.44 in polar and apolar energy per area (Table 4).Enzyme-substrate structural strength considering the number of intermolecular hydrogen bonds are 238, 240, and 240 for the 2BOQ-lignin, 1YYD-lignin, and 1LLP-lignin sequentially. As the lowest number of hydrogen bonds, which showed maximum stability, 2BOQ was the significant one. 

**Table 2.** Formation of enzyme-substrate complexes following molecular docking, where the binding affinity scale (kcal/mol) refers to the intensity of docking stability between the enzymes and substrates individually. The upper and lower values of RMSD (*Å*) are regulated with the fluctuations of their corresponding binding affinity values.

Enzymes	Substrates	Binding affinity (kcal/mol)	RMSD (*Å*)	
			UB (*Å*)	LB (*Å*)
1LLP	Lignin (Organosolv)	−7.00	2.692	1.707
1YYD	Lignin (Organosolv)	−6.50	14.336	3.949
2BOQ	Lignin (Organosolv)	−8.00	3.569	1.938
1HOB	Cellulose (Gel)	−6.00	9.181	3.39
3GZK	Cellulose (Gel)	−5.30	7.605	3.017
1OA9	Cellulose (Gel)	−6.70	9.644	4.388
Root mean square deviation (RMSD); Upper bound (UB); Lower bound (LB). 1LLP and 1HOB were taken as standard enzymes for hydrolyzing lignin and cellulose, respectively.				

### Catabolism of the cellulose-degrading enzymes based on structural interactions 

The binding affinity of the complex between “1OA9-cellulose” was found to be more significant (−6.7 kcal/mol) than the “3GZK-cellulose” and “1H0B-cellulose” complexes, with energy scales of −5.3 and −6.0 kcal/mol, respectively (Table 2). Tyr163 (3.05), Glu124 (2.71), Trp26 (3.14), and Glu207 (3.19, 3.04, and 2.93) hydrogen bond interactions form complex 1H0B-cellulose (Fig. 3A). Simultaneously, complex hydrophobic networking is developed by cellulose with the neighboring amino acid residues (Pro137, Gly138, Trp159, Trp161, Trp108, Asp106, Tyr59, Trp128, Met126) of the enzyme (Table 3). Similarly, the complex between “3GZK-cellulose” (Fig. 3B) is mainly a network of hydrogen bonds with the residues- Arg410 (3.17 and 3.32 Å), Asp398 (3.29 Å), Asp345 (2.84 Å), Trp401 (2.94 Å), Gly342 (2.97 Å), and hydrophobic interactions with Glu340, IIe400, Ala344, Met4005, Trp343, and Val406 (Table 3). Asp10 (3.18), Leu130 (2.98), Val128 (3.05), and other residues (Tyr146, Gly127, Gly129, Tyr8, Tyr110, Gly112, Thr6, and Asn178) (Table 3).

Representative RMSD values of cellulose gel complexes complexed with 1HOB, 3GZK, and 1OA9 were between 0.6 and 1.20, with the 1OA9-cellulose complex showing a higher fluctuation rate within the 30 to 45 ns time period than the other complexes (Fig. 4B). The enzyme-substrate complexes’ MolSA resulted in values ranging from 9,138.552 to 22,534.41 Å^2^ (Fig. 4C). The RMSF values for the enzymes’ flexibility assessment did not exceed 2.4 Å (except 3GZK), referring to a more stable enzyme structure with fewer beta-sheet and alpha-helix regions. The enzyme-3GZK possesses an RMSF value of 3.7 Å that the protein structure is less stable and bears more α-helix and/or β-sheet regions resulting from the dynamic simulation (Fig. 5B). Among the three complexes, the 1OA9-cellulose gel complex showed a more compact and rigid structure following the value of 15.80 collected from the Rg output data. Besides, the other complexes showed values of 23.20 (3GZK-Cellulose gel) and 16.50 (1H0B-Cellulose gel) that are less significant than the former complex (Fig. 5C). The SASA profiling conformed to the expansion of the “1OA9-cellulose gel” complex surface area during the 50 ns dynamics simulation process as more significant than the other complexes, representing an area size of 84,002, whereas the enzyme complexes’ “1H0B-cellulose” and “3GZK-Cellulose” documented comparatively higher SASA values, respectively. It is important to recommend that the lowest SASA value of the system display more significant results with the 1OA9-cellulose gel complex (Fig. 5D). Amongst all the enzymes, 1OA9 had the lowest polar energy per area (3,340.94). On the contrary, 1H0B had the lowest apolar energy generated per 2 area (3,340.94). 3GZK demonstrated stark contrast, as it had the largest difference between the polar and apolar values, 7,632.39 and 11,981.65, respectively (Table 4). The intermolecular hydrogen bonds are 160, 180, and 400 for the 1OA9-Cellulose, 1H0B-Cellulose, and 3GZK-Cellulose complexes, respectively. The lowest number of hydrogen bonds showed a more stable complex formation of cellulose with 1OA9 than with all other enzymes.

**Table 3.** Quantitative measurement of the existence of hydrophobic bond and H-bond interactions between each of the enzyme-substrate complexes precisely.

Enzymes	Substrates	Amino acid interactivity	
		H-bond interactions	Hydrophobic interactions
1LLP	Lignin (Organosolv)	Pro339 (2.70 *Å*), Pro296 (3.00 *Å*), Asp201 (2.72 *Å*), Asn119 (3.15 *Å*), Cys34 (2.89 & 3.09 *Å*), Gln33 (2.95 *Å*), Pro340 (3.19 *Å*) Glu37 (3.29 *Å*) and Lys342 (3.19 and 3.09 *Å*)	Ala36, IIe338, Ser195, Ala336, Val335, Leu328, Phe303, IIe295, Thn196, IIe199, Gly198, Pro121, His341, His30
1YYD	Lignin (Organosolv)	Gln200 (2.92 *Å*), Gln302 (3.02 *Å*), Thr300 (2.97 *Å*), Asp198 (3.16 *Å*), Asn31 (3.34 *Å*), Gln330 (3.12 *Å*), Pro342 (2.81 *Å* and 2.78 *Å*) and Asp343 (3.04 *Å*)	Pro303, Thr196, Pro118, Phe195, Lys297, Pro298, Glu32, Ala339, Gly344, Ser336, Ser192, Thr193
2BOQ	Lignin (Organosolv)	Lys215 (3.15 and 3.14 *Å*), Vall77 (3.11 *Å*), His136 (2.96 and 2.72 *Å*), Asp135 (2.72 and 2.88 *Å*), IIe71 (2.78 *Å*), Ala77 (2.74 *Å*) and His39 (2.87 *Å*)	Alal73, Alal72, His169, Leu228, Asn214, Lys176, Glu140, Pro76, Phe142, Asp82, Gly80, Asn74, Ala79, Arg43, His47, Phe186
1H0B	Cellulose (Gel)	Tyr163 (3.05 *Å*), Glu124 (2.71 *Å*), Trp26 (3.14) and Glu207 (3.19, 3.04 and 2.93 *Å*)	Pro137, Gly138, Trp159, Trp161, Trp108, Asp106, Tyr59, Trp128, Met126
3GZK	Cellulose (Gel)	Arg410 (3.17 and 3.32 *Å*), Asp398 (3.29 *Å*), Asp345 (2.84 *Å*), Trp401 (2.94 *Å*), Gly342 (2.97 *Å*) and Asn409 (3.11 *Å*)	Glu340, IIe400, Ala344, Met4005, Trp343, Val406
1OA9	Cellulose (Gel)	Arg7(2.70 *Å*), Gly147 (2.90 *Å*), Phe131(2.98 *Å*), Asp10 (3.18 *Å*), Leu130 (2.98 *Å*), Val128 (3.05 *Å*) and Asp120 (2.83)	Tyr146, Gly127, Gly129, Tyr8, Tyr110, Gly112, Thr6, Asn178

Hydrogen bond (H-bond).

Considering all the MDS profiles like RMSD, RMSF, SASA, MolSA, Rg, and hydrogen bonds, the catabolic significance of substrate-degrading enzymes in the current research has resulted in 2BOQ <1LLP <1YYD. Similarly, the catabolic profiles of the cellulose-degrading enzymes resulted in 1OA9 <1H0B < 3GZK. The consequence of cellulose degradation is the production of glucose and xylose, which are simple forms of sugar and are edible for human beings as an energy source.

## Discussion

Food production, preservation, and supply for the ever-increasing global population have become a matter of great challenge, where alternative edible sources of food and energy are desperately needed. The use of non-food lignocellulose biomass can be a good option for producing edible sugars for energy if they are properly manipulated [32,33]. The catalytic activity of the enzymes can play a significant role in the lignocellulose manipulation procedure, where the lignin-degrading enzymes first degrade the lignin wall to expose cellulose and hemicellulose [34,35]. Afterward, the cellulose microcrystals are hydrolyzed with the cellulose-degrading enzymes to produce glucose and xylose, which are completely edible for human beings [36]. Considering all those things, in this current research, lignin (PubChem CID-73555271) and cellulose gel (PubChem CID-14055602) were used as the main substrates on which the catabolic activity of enzymes was studied depending on their enzyme-substrate complexing characteristics, inter-molecular interactions, and MDS properties. It’s very pivotal to investigate the active sites of enzymes to conduct point specific super docking instead of conventional docking, which was undertaken by this researcher using the high-throughput tool COACH-D and its algorithm [14,15]. This resulted in a sufficient number of amino acid residues with enormous binding energies (kcal/mol) (). To study lignin hydrolysis activity—manganese peroxidase (PDB ID-1YYD), versatile peroxidase (PDB ID-2BOQ), and lignin peroxidase (PDB ID-1LLP) enzymes were used , whereas Acidocaldarius cellulase (PDB ID-3GZK), Melanocarpus albomyces endoglucanase (PDB ID-1OA9) , and standard endoglucanase cel12A (PDB ID-1H0B) were used for cellulose degradation. 

To understand the catabolic activity of the enzymes toward lignin and cellulose substrates, depending on their binding affinity and RMSD values, molecular docking was conducted individually [42] following the optimization of all the substrates and enzymes. The “2BOQ-lignin” and “1OA9-cellulose gel” resulted in the best fitting scores of −8.00 and −6.7 (kcal/mol), respectively (Table 2). The docking score detects comparatively selective, potent, and efficient candidates for surface modeling [43]. Moreover, for the analysis of the enzyme-substrate interactions, to understand the hydrophobic and hydrogen bonding interactions (Table 3) between each of the enzyme-substrate complexes, their quantitative networking model was developed using “Java interface” of computer programming (Figs. 2 and 3). MDS has proven to be the best authentic method for investigating biomolecular interactions, evaluating the atomic level’s stability and the resulting output data of the dynamic trajectories, which paves the way for the quantitative relationship between enzyme and substrate structure and functions [44]. In this current study, the YASARA (version 11.9.18) dynamics simulator was conducted, maintaining all the physio-chemical and physiological parameters (Temperature—310K, pH—7.4; 0.9% salt concentration; addition of ions like Na+ and Cl−, etc.) during the 50 ns simulation run time [45], and the output data resulted from the dynamics state of the enzyme’s capability to catabolize the lead substrates (Lignin, Cellulose gel). The enzyme-substrate binding energy was evaluated using the equation of YASARA [24]

**Table 4.** SASA of the enzyme-substrate complexes obtained from MDS for 50 ns.

Enzymes	Substrates	WPR (*Å*)	GIC	TNR	Total Area/Energy		
					Polar	Apolar	UNK
1LLP	Lignin (Organosolv)	1.400	No	343	4,592.05	9,123.59	0.0
1YYD	Lignin (Organosolv)	1.400	No	357	5,226.47	9,473.63	0.0
2BOQ	Lignin (Organosolv)	1.400	No	319	4,401.29	8,374.44	0.0
1HOB	Cellulose (Gel)	1.400	No	227	4,268.03	5,028.56	0.0
3GZK	Cellulose (Gel)	1.400	No	532	7,632.39	11,981.65	0.0
1OA9	Cellulose (Gel)	1.400	No	208	3,340.94	5,337.87	0.0

Water probe radius (WPR); Gradient in calculation (GIC); Total No. of residues (TNR); Unknown areas (UNK).

Δ*G* = Δ*G*vdW + Δ*G*Hbond + Δ*G*elec + Δ*G*tor + Δ*G*desol

where, 

Δ*G*elec = electrostatic term for docking energy; 

Δ*G*vdW = van der Waals term for docking energy; 

Δ*G*desolv = desolvation term for docking energy

Δ*G*Hbond = H bonding term for docking energy; 

Δ*G*tor = torsional free energy term for the compound when the compound transits from unbounded to bounded state; 

The RMSD values of the lignin-degrading 1LLP, 1YYD, and 2BOQ enzymes were 1.25, 1.30, and 1.10 Å sequentially (Fig. 4A). On the other hand, the RMSD values were 1.20, 0.80, and 0.75 Å for 1H0B, 3GZK, and 1OA9, respectively (Fig. 4B). The finding relates the catabolic properties of the enzymes with the other established findings [46,47]. 

To analyze the RMSD values (for frame “*x*”), the following algorithm was used- 


$${\mathrm{RMSD}}_{X}=\sqrt{\frac{1}{N}\sum_{i=1}^{N}(r_{i}^{l}(t_{x}\mathrm{))}-r_{i}(t_{\mathrm{ref}}{\mathrm{))}}^{2}}$$

Here, “*N*” is the number of atoms in the atom selection; “*t*ref” is the reference time (typically the first frame is used as the reference, and it is regarded as time, “*t* = 0”); and “*r*” is the position of the selected atoms in frame “*x*” after superimposing on the reference frame, where frame *x* is recorded at the time : “*t*x.” The procedure is repeated for every frame in the simulation trajectory. 

There are two types of available MolSA calculations, namely- vdW and solvent accessible [48]. More commonly, the enzyme-substrate complex stability should be increased with the decrease of the surface area (Å^2^) [49]. Considering the criteria, 2BOQ-lignin and 1OA9-cellulose gel complexes were the more stable ones, with 15,202.688 and 9,138.552 Å^2^, respectively (Fig. 4C). In the same way, the fluctuation of all the complexes resulted below 2 Å (Fig. 5A and B) except the “3GZK–cellulose gel” complex (Fig. 5B), from the RMSF values of the 50 ns MDS. RMSF data represent the flexibility and strength of enzyme-substrate complexes (Fig. 5). The algorithm used for the characterization of local changes along the enzyme chain-


$${\mathrm{RMSF}}_{i}=\sqrt{\frac{1}{T}\sum_{t=1}^{T}(r_{i}^{l}(t_{\;}\mathrm{))}-r_{i}(t_{\mathrm{ref}}{\mathrm{))}}^{2}}$$

Here, “*T*” is the trajectory time over which the RMSF is calculated, “*t*ref” is the reference time (usually for the first frame, and is regarded as the zero of time); “*r*” is the position of atom “*i*” in the reference at the time “*t*ref”, and “*r*” is the position of the atom “*i*” at the time “*t*” after superposition on the reference frame. 

The Rg represents the tightness of the protein structure, where a decreased fluctuation level indicates the increased firmness and rigidity of the system [21]. Among the six complexes, “2BOQ-lignin” and “1OA9-cellulose gel” complexes showed the more compact values means 19.50 and 15.80 nm, respectively (Fig. 5C). The SASA uses parameters to check the changes in the arrangement of the enzyme-substrate complexes retrieved from the molecular docking and MDS to figure out the efficacy of the interaction between the enzyme and substrate. It detects the interactions occurring between the complex surface and the water molecules based on the total energy per area of the substrate and the compound [50]. Higher SASA values indicate unstable structures due to increased contact between the hydrophobic amino acids with the water [51]. Contact between the hydrophobic part and the water is highly unfavorable, destabilizing the interaction between the enzyme and substrate. The interaction between lignin and 1YYD was highly unfavorable due to their higher total energy per area. At the same time, 2BOQ-lignin scored the lowest total energy per area (Table 4), causing them to have the strongest interaction [52]. Similarly, the “1OA9-cellulose gel” complex possesses the lowest total energy per area, ensuring their highest interaction profile than the others (Fig. 5D). It is important to mention that enzymes in both the lignin as well as Cellulose Gel had zero value under unknown. This is because the dynamic simulation conducted was accurate, as no unknown residue was found [53]. The enzyme-substrate complexes’ conformational stability was determined via the number of hydrogen bonds retrieved from the 2BOQ-lignin (238) and 1OA9-Cellulose gel (160) complexes.

This *in silico* study focuses on the catabolic activity of numerous industrial enzymes in the degradation of non-food lignocellulose biomass into simple sugars (glucose, xylose) based on enzyme-substrate interactions and structure-based molecular networking. This cutting-edge method for biosynthesizing food energy from completely non-food sources by modulating enzyme bioconversion activity could be a revolutionary option for securing a hunger-free world for future generations, which is the primary goal of this research. 

## Conclusion

The catabolic activity of six enzymes was examined *in silico* to configure their efficacy in degrading lignocellulose biomass into functional sugars as food energy, as a part of a study of alternate food supplies for the future to alleviate hunger. The designed enzymes were optimized using energy minimization and applied to molecular docking on lignin and cellulosic components, with a 50 ns runtime of the MDS, resulting in RMSD, MolSA, RMSF, SASA, Rg, and Hydrogen bond amount values in the favorable ranges, indicating the catabolic strength of the enzymes of interest. Using all parameters of docking and dynamics simulations, the researchers discovered that Versatile Peroxidase and Melanocarpus albomyces endoglucanase enzymes have the most efficient features for bioconverting lignin and cellulose microcrystals, respectively. Researchers have had a lot of trouble figuring out which biocatalysts are appropriate for the saccharification process before fermentation. This research gives an authentic means of monitoring and predicting prospective enzyme activity for a successful hydrolytic biological conversion process before doing so directly in the fermenter to produce edible sugars at that time. The destroyed lignin structure exposes the cellulosic contents, which are eventually converted into glucose and xylose as edible sugars for humans, who are in desperate need of ways to alleviate the global food crisis, produce biofuels, and manage agricultural waste. This study will aid the next generation of scientists, particularly bioinformatics and bioengineering experts, in pre-examining the catalytic potentialities of their enzymes of interest for a successful biological reaction before conducting it inside any bioreactor system. Agricultural waste materials can be anticipated to produce a variety of value-added goods, ranging from basic food components to carbon-neutral biofuel generation, using this type of *in silico* technique.

## List of Abbreviations

COACH-D, Core algorithm; MDS, Molecular dynamic simulation; MolSA, Molecular surface area; Ns, Nanoseconds; PDB, Protein Data Bank; PSA, Polar surface area; Rg, Radius of gyration; RMSF, Root mean square fluctuation; RMSD, Root mean square deviation; SASA, Solvent-accessible surface area; UCSF Chimera, University of California San Francisco Chimera; YASARA, Yet another scientific artificial reality application; LARMD, Ligand and Receptor Molecular Dynamics; B-Facator, Debye-Waller factor; CID, Compound identification number.
